# Measuring Partnership Activities: Partnerships in Environmental Public Health Evaluation Metrics Manual

**DOI:** 10.1289/ehp.1205512

**Published:** 2012-07-02

**Authors:** Christina H. Drew, Kristianna G. Pettibone, Liam R. O’Fallon, Gwen W. Collman, Linda S. Birnbaum

**Affiliations:** Division of Extramural Research and Training, National Institute of Environmental Health Sciences, National Institutes of Health, Department of Health and Human Services, Research Triangle Park, North Carolina, E-mail: drewc@niehs.nih.gov; Director, National Institute of Environmental Health Sciences and National Toxicology Program, National Institutes of Health Department of Health and Human Services, Research Triangle Park, North Carolina, E-mail: birnbaumls@niehs.nih.gov

The National Institute of Environmental Health Sciences (NIEHS) has had a long-standing commitment to facilitate and engage community groups in environmental health science research. In 2008, the NIEHS established the Partnerships for Environmental Public Health (PEPH) program to formalize our commitment to outline a coordinated vision for community and academic partnerships. Since then, > 400 grantees have participated in activities designed to foster net-working among grantees within the various NIEHS programs, including webinars and workshops on communicating PEPH findings and translating research to public health policy.

A key tenet of the PEPH is community engagement. In response to an NIEHS Request for Information in 2008, the community shared concerns about the lack of evaluation capacity and the need for tools and approaches to develop project specific evaluation metrics for public health–related program areas. In response, the NIEHS developed the *PEPH Evaluation Metrics Manual* (NIEHS 2012b) with significant input from PEPH grantees, program staff, and experts in the field, including input from > 250 individuals at > 30 professional meetings.

Evaluation of PEPH programs provides useful bene-fits to grantees, including the ability to *a*) identify program successes; *b*) determine whether a project worked and why (or why not); *c*) identify areas for program improvement and increased efficiency; *d*) describe expenditures and justify a need for additional funding; *e*) recognize and respond to public needs and wants; *f* ) identify new audiences and applications for projects; and *g*) prioritize research and plan for the future. Evaluation also may help grantees find allies in other agencies, services, or sectors; publicize achievements in communities; and inform policy and other decision making. Evaluation metrics also provide a means for the NIEHS to evaluate the success of individual projects and the PEPH program as a whole.

Typical approaches to evaluating research outcomes involve analyzing publications. However, because many PEPH programs do not publish findings related to their community engagement, we worked with grantees and community members to identify appropriate metrics to measure and demonstrate success in five areas that are common to many PEPH grantees:

- Partnering (working with other organizations to conduct environmental public health activities)
- Leveraging (using the resources already available to a project to obtain additional resources)
- Disseminating findings (providing information about environmental public health issues and results of PEPH research)
- Training (developing programs that teach researchers, community members, workers, students, and others strategies for reducing hazardous environ-mental exposures)
- Capacity building (performing activities that improve an organization’s ability to achieve its mission).

For each of these five areas, the NIEHS developed an illustrative logic model to demonstrate connections among project activities, outputs, and impacts; > 80 examples of metrics for each activity, output, and impact are provided as examples for grantees developing metrics for evaluating the progress and achievements of their own programs.

We present the partnership logic model (Figure 1; NIEHS 2012b) to illustrate how metrics can be developed from a logic model. When developing program logic models, it can be helpful to begin by working with partners to identify and articulate the desired impacts of the program. Once partners agree about “where they want to go” (impacts), discussions of “how to get there” (activities) naturally follow. Then partners can determine “how will we know we are there?” (outputs and metrics). The nouns, adjectives, verbs, and adverbs in the answers to these questions then serve as the basis for metrics.

Examples of metrics from grantee programs that address components of the partnership logic model include:

- Demonstrating success at identifying partners (activity 1), the University of Cincinnati’s anti-idling campaign provided a description of the partners involved and the resources they bring to the project: Cincinnati Public Schools provided access to students and schools; the Cincinnati Health Department provided nursing services; a councilwoman provided credibility and the ability to attract attention to the project; and the Hamilton County Department of Environmental Services provided training and information to Cincinnati Public School staff and students.
- Demonstrating their success in involving community partners in research, the University of Florida, the Farmworker Association of Florida (FWAF), and Best Start Inc. (a social marketing research firm) described how partners were involved in the Together for Agricultural Safety Project (output 4). Members of the FWAF helped develop and lead focus groups and provided input into the survey development; researchers from the University of Florida and Best Start Inc. collected data; and 382 farm-workers provided data. All three partners also contributed to an article summarizing the process by which the project was implemented (Flocks et al. 2001).
- The Detroit Community-Academic Urban Research Center is a collaborative partnership that includes the University of Michigan Schools of Public Health, Nursing, and Social Work; the Detroit Department of Health and Wellness Promotion; eight community-based organizations; and the Henry Ford Health System. To demon-strate their success at expanding research collaborations, they provided details of the additional research opportunities that were generated as a result of the partnership (impact 3). These research opportunities included four grants from the NIEHS: $5 million for establishing a Children’s Environmental Health Sciences Center; $2.4 million to conduct the Community Organizing Network for Environmental Health; $2.5 million for a household intervention to reduce asthma triggers; and $2.5 million for an epidemiologic project to characterize the effect of roadway-associated air pollution on the exacerbation of asthma in children.

PEPH grantees are encouraged to adapt metrics to fit the unique characteristics of their communities, and those with multi-site initiatives may want to identify metrics that are applicable for all sites. Although the *PEPH Evaluation Metrics Manual* was designed with PEPH grantees in mind, it may be useful to community groups, advocates, and others working to address environmental public health issues. The principles outlined in the manual also may be useful to those interested in measuring the success of basic research programs.

We anticipate that the PEPH manual will be a living document that we will update periodically. Opportunities for expansion include new evaluation topics (e.g., cost–benefit analyses and econometric evaluations), as well as new approaches used in programs (e.g., social media) and new examples of metrics drawn from the ever-expanding network of PEPH grantees.

Program staff have been conducting training related to evaluation metrics and developing stand-alone materials that will be publically available through the PEPH website (NIEHS 2012a). NIEHS staff is available to conduct webinars related to the *PEPH Evaluation Metrics Manual*. We encourage those interested in learning more about the manual or about developing metrics for their program to explore the manual and contact the authors with any questions or concerns.

## References

[r1] Flocks J, Clarke L, Albrecht S, Bryant C, Monaghan P, Baker H. (2001). Implementing a Community-Based Social Marketing Project to Improve Agricultural Worker Health.. Environ Health Perspect.

[r2] NIEHS (National Institute of Environmental Health Sciences). 2012a. Partnerships for Environmental Public Health (PEPH). Available: http://www.niehs.nih.gov/peph [accessed 11 June 2012].

[r3] NIEHS (National Institute of Environmental Health Sciences). 2012b. Partnerships for Environmental Public Health Evaluation Metrics Manual. NIH Publication No. 12-7825. Available: http://www.niehs.nih.gov/pephmetrics [accessed 13 March 2012].

